# Isolated Proteinuria in Uncomplicated Twin Pregnancies vs. Singleton Pregnancies; Measured by Spot Protein‐Creatinine Ratio

**DOI:** 10.1155/jp/4012487

**Published:** 2026-08-28

**Authors:** Zahra Naeiji, Shirin Ghalehtaki, Sanaz Mahdi Nejad, Sahar Aslani, Sahar Mohammadpour Doroudkhani

**Affiliations:** ^1^ Department of Obstetrics and Gynecology, School of Medicine, Mahdiye Hospital, Shahid Beheshti University of Medical Sciences, Tehran, Iran, sbmu.ac.ir; ^2^ Department of Obstetrics and Gynecology, School of Medicine, Shahid Beheshti University of Medical Sciences, Tehran, Iran, sbmu.ac.ir; ^3^ School of Medicine, Tehran Medical Branch, Islamic Azad University, Tehran, Iran, tiau.ac.ir; ^4^ General Practitioner, Rasht Health Network, Guilan University of Medical Sciences, Rasht, Iran, gums.ac.ir

**Keywords:** pre-eclampsia, pregnancy, proteinuria

## Abstract

**Background and Objectives:**

Proteinuria is used for prediction of pre‐eclampsia; however, the cutoff values are suggested for singleton pregnancies and might not be appropriate for twin pregnancies. In this study, we examined whether the urine spot protein‐creatinine ratio (SPCR) is higher in twin than singleton pregnancies and what factors are associated with this difference.

**Materials and Methods:**

In this prospective longitudinal study with repeated measures, pregnant mothers (aged 15–45 years) who referred to Mahdiye Hospital, Tehran, Iran, from April 2021 to November 2022 for routine prenatal care at least from the 15th gestational week were included in the study. Maternal age, body mass index, and gravidity were recorded. For measurement of SPCR, spot urine samples were collected from each participant at three predefined time points during pregnancy. Pregnancies complicated with (pre)gestational diabetes or pre‐eclampsia, urinary tract infection, or other renal diseases were excluded from the study. The within‐subject effects of variables on the groups were evaluated using repeated‐measures ANOVA. A *p* value < 0.05 was considered statistically significant.

**Results:**

There were 89 mothers in the singleton group and 70 in the twin group. The mean age between the groups was not significantly different, but BMI and the distribution of gravidity differed between them. Higher median urine SPCR values were found in twin pregnancies in the mid‐second and third trimesters (*p* = 0.005 and 0.022, respectively), with a significant trimester‐by‐group interaction effect in the overweight (*p* = 0.008) and obese subgroups (*p* = 0.004), as well as in women with Gravidity 1 (*p* = 0.012) and Gravidity ≥ 3 (*p* = 0.018).

**Conclusion:**

Twin pregnancies have higher urine SPCR values than singletons; these findings suggest that current cutoff values may need re‐evaluation in future studies to improve the prediction of pre‐eclampsia in multifetal gestations.

## 1. Introduction

Pregnancy is an important part of every woman′s life and having an uneventful pregnancy/delivery is important for fetal and maternal health. Pre‐eclampsia is a multisystem pregnancy disorder that results in placental malperfusion and release of soluble factors into the circulation; this will, in turn, result in maternal vascular endothelial injury and lead to hypertension and multiorgan injury [1, 2]; the injury to organs, especially the kidneys, results in pre‐eclampsia or eclampsia that may complicate the pregnancy, cause early delivery and stillbirth, and also endanger the woman′s life after delivery [3].

As there are no curative treatments for pre‐eclampsia, except for delivery, it is important to predict this condition and prevent its occurrence or reduce its severity. Accordingly, algorithms have been suggested for each trimester of pregnancy to perform clinical tests and measure the biochemical markers for prediction of pre‐eclampsia in the pregnant woman [4–6]. These include routine measurement of the mother′s blood pressure and serum/urine tests, including urate, alanine transaminase, and proteinuria. Specific biomarkers, like placental growth factor, are also suggested to be measured after estimating the risk of pre‐eclampsia [5, 7].

Urine spot protein‐creatinine ratio (SPCR) is suggested as an appropriate tool for measurement of abnormal protein concentration since more than 40 years ago [8] and for prediction of pre‐eclampsia since 2001 [9]. The International Society for the Study of Hypertension in Pregnancy (ISSHP) has suggested the cutoff value for SPCR to be 300 mg/mmol (0.3 mg/mg) to predict pre‐eclampsia [10], and evidence has confirmed it as a sensitive tool (> 90%) at the specified cutoff [11]. Others have suggested 350 mg/mmol (3.5 mg/mg) to confirm nephrotic proteinuria and > 20 mg/mmol (0.2 mg/mg) to detect proteinuria [12] with failure to identify pre‐eclampsia only in 6% of women [13]. Others have suggested SPCR of > 36 mg/mmol (0.36 mg/mg) 86% sensitive and 100% specific for diagnosis of pre‐eclampsia [14]. However, the cutoff values suggested are for singleton pregnancies and might not be appropriate for multifetal pregnancies, as they might have different values. Furthermore, National Institute for Health and Care Excellence (NICE) and American College of Obstetricians and Gynecologists (ACOG) guidelines have suggested multifetal gestation as a risk factor of pre‐eclampsia [15]. Therefore, it is necessary to pay greater attention to prediction of pre‐eclampsia in mothers with multiple fetuses. However, to date, no guidelines have addressed this issue.

Previous studies have also documented that women with twin pregnancies have a higher urine SPCR value [16–18]. Therefore, it may be necessary to modify the urine SPCR cutoff values for twin pregnancies, while this has not been recommended by the guidelines yet. Another matter to be considered is the dependence of SPCR on ethnicity. Bhatti et al. [19] suggested the different detection rates in African–American and Caucasian pregnant women (considering the suggested cutoff of 30 mg/mmol). Therefore, it is necessary to determine the appropriate cutoff in each population separately; notably, it has not been evaluated for Iranian women. Therefore, in the present study, our first goal was to compare the urine SPCR values between twin and singleton uneventful pregnancies in a sample of Iranian population and find whether twin pregnancies have higher urine SPCR values than singleton pregnancies; our second goal was to find what factors are associated with this difference.

## 2. Materials and Methods

In this prospective longitudinal observational study with repeated measures, pregnant mothers who referred to Mahdiye Hospital, Tehran, Iran, from April 2021 to November 2022 for routine prenatal care at or before 15 weeks of gestation were screened for eligibility, based on the inclusion criteria: age 15–45 years, referring at 15 weeks of gestation or earlier. The study protocol was approved by the Ethics Committee of Shahid Beheshti University of Medical Sciences (Code: IR.SBMU.MSP.REC.1400.200).

The sample size was calculated at 76 in each group, considering the value of urinary Pr/Cr of 0.16 in singleton pregnancy and 0.21 in twin pregnancy, according to the study by Smith et al. [16], study power of 80% and error Type I at 5%. A total of 166 mothers were initially included (76 twin and 90 singleton pregnancies). Participants who developed pre‐eclampsia during follow‐up were excluded from the final analysis to ensure inclusion of only uneventful pregnancies. After exclusion of one singleton and six twin pregnancies, 159 participants remained for final analysis, including 89 singleton and 70 twin pregnancies. Mothers who had the inclusion criteria were selected consecutively (nonrandomized) and included in the study by convenient sampling method; the researcher explained the study protocol and objectives to the participants and asked them to read and sign the written informed consent. Mothers with renal or autoimmune disease or chronic diseases were not enrolled in the study.

Maternal age, body mass index (BMI), and gravidity were recorded. BMI categories were defined according to the World Health Organization criteria as normal weight (18.5–24.9 kg/m^2^), overweight (25–29.9 kg/m^2^), and obese (≥ 30 kg/m^2^). All eligible participants were followed throughout their pregnancy. As per the study protocol, repeated spot urine samples were collected from the same individual at three predefined time points: early second trimester (16–20 weeks), mid‐second trimester (24–28 weeks), and third trimester (34–38 weeks) to measure the SPCR.

Pregnancies complicated with (pre)gestational diabetes, pre‐eclampsia, and urinary tract infection were excluded from the study. Pre‐eclampsia was defined according to ACOG criteria as new‐onset hypertension after 20 weeks of gestation (systolic blood pressure ≥ 140 mmHg or diastolic blood pressure ≥ 90 mmHg) with proteinuria or, in the absence of proteinuria, new‐onset thrombocytopenia, renal insufficiency, impaired liver function, pulmonary edema, or cerebral/visual symptoms.

### 2.1. Statistical Analysis

The collected data were input into the statistical software IBM SPSS Statistics for Windows Version 21.0 (IBM Corp. 2012. Armonk, New York: IBM Corp.), used for statistical analysis. Descriptive results were presented by frequency (percentage) for categorical variables. Normally distributed continuous variables were presented as mean ± standard deviation (SD), whereas nonnormally distributed continuous variables were presented as median (IQR). Shapiro–Wilk was used to evaluate the normal distribution of the numeric variables. Although some variables showed nonnormal distribution, repeated measures ANOVA was used due to its robustness to moderate deviations from normality. To evaluate changes in SPCR levels across trimesters, within‐subject analyses were performed using repeated‐measures ANOVA. Sphericity was assessed via Mauchly′s test, and the Greenhouse–Geisser test was used for investigating the within‐subject effect of variables on the groups (trimester and BMI), when the sphericity assumption was violated. For between‐group comparisons at each time point, the Mann–Whitney *U* test was used. *p* values < 0.05 were considered statistically significant.

## 3. Results

During the study, one singleton and six twin pregnancies were excluded due to pre‐eclampsia. Ultimately, a total of 89 singleton and 70 twin pregnancies completed the study, and SPCR measurements were obtained for all participants at all the predefined time points, with no missing data. Comparing the baseline variables of the groups showed no difference in mean age of the groups, but there was a significant difference between the groups in mean BMI (*p* = 0.004); mean BMI of the mothers with singleton pregnancy was higher than those with a twin pregnancy (Table 1). About one‐third of the participants had Gravidity 1 (maximum gravidity was six); there was a significant difference in the frequency of gravidity between singleton and twin pregnancies (*p* = 0.022; Table 1). All mothers had normal blood pressure.

**Table 1:** Comparison of the baseline characteristics of the study groups.

Variable	Categories	Total	Singleton pregnancy (*N* = 89)	Twin pregnancy (*N* = 70)	*p*
Age (years), mean ± SD		30.46 ± 6.13	30.77 ± 5.99	30.07 ± 6.33	0.474 ^∗^
BMI (kg/m^2^), mean ± SD		29.84 ± 4.86	30.82 ± 5.40	28.60 ± 3.78	0.004 ^∗^
Gravidity, *N* (%)	Gravidity 1	53 (33.3)	26 (29.2)	27 (38.6)	0.022 ^∗∗^
	Gravidity 2	51 (32.1)	24 (27)	27 (38.6)	
	Gravidity ≥ 3	55 (34.6)	39 (43.8)	16 (22.9)	

^∗^The results of independent samples *T*‐test.

^∗∗^Results of chi‐square test.

**Figure 1:**
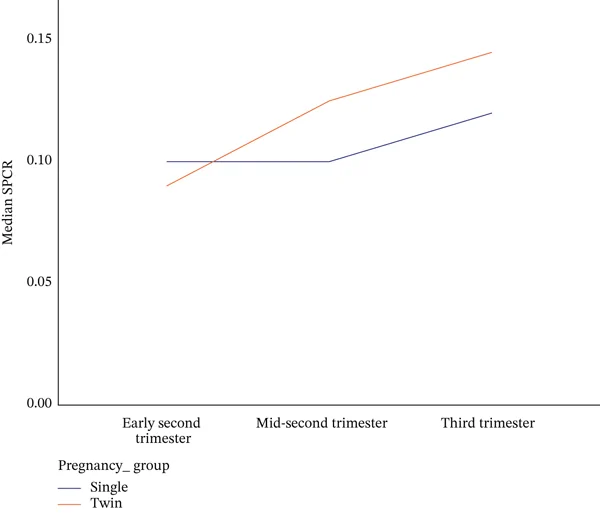
The trend of changes in median SPCR in the two study groups.

Median values of urine SPCR are shown in Table 2; as shown, no significant difference was observed in the early second trimester; whereas in the mid‐second and third trimesters, a significant difference was observed in median urine SPCR between singleton and twin pregnancies; higher median values were found in twin pregnancy (*p* = 0.005 and 0.022, respectively). The results of multivariate test showed a significant interaction effect of trimester‐group (*p* < 0.001) (Figure 1).

**Table 2:** Comparison of the urine SPCR in the three trimesters between the study groups.

	Variable	Total	Singleton pregnancy (*N* = 89)	Twin pregnancy (*N* = 70)	*p* ^∗^
Early second trimester	Median (IQR)	0.090 (0.070–0.140)	0.100 (0.070–0.140)	0.090 (0.060–0.153)	0.836
Mid‐second trimester	Median (IQR)	0.110 (0.075–0.150)	0.100 (0.075–0.150)	0.125 (0.080–0.190)	0.005
Third trimester	Median (IQR)	0.120 (0.080–0.170)	0.120 (0.080–0.170)	0.145 (0.090–0.200)	0.022

^∗^The results of Mann–Whitney *U* test. Results are presented as median (IQR). All values are presented as protein‐to‐creatinine ratio (mg/mg).

**Figure 2:**
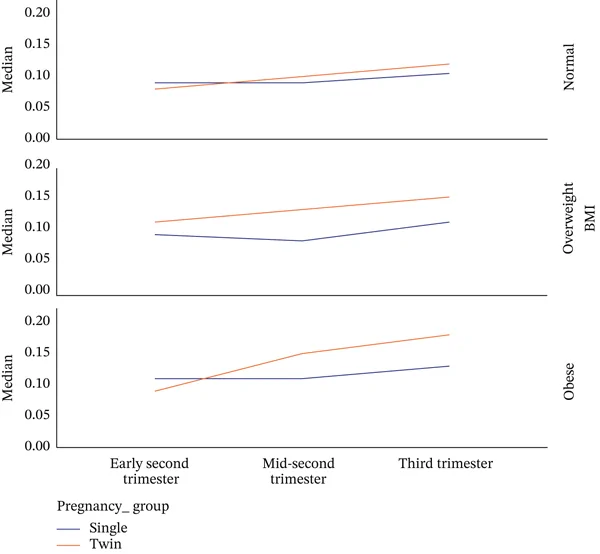
The trend of changes in median SPCR in the two study groups based on BMI categories.

Then, we separated the results based on BMI categories and the results showed significant interaction effect of trimester‐group in the overweight (*p* = 0.008) and obese subgroups (*p* = 0.004), but not in the normal BMI group (*p* = 0.453). Median urine SPCR was not different between singleton and twin pregnancies in the early second trimester in any BMI categories. In the mid‐second and third trimesters, overweight women with twin pregnancies had a higher median urine SPCR (*p* = 0.003 and 0.010, respectively), whereas normal weight and obese women did not (*p* > 0.05; Table 3, Figure 2). Although interaction effects were significant, pairwise comparisons did not consistently show significant differences in median SPCR between singleton and twin pregnancies across all BMI categories.

**Table 3:** Comparison of the urine SPCR in the three trimesters between the study groups based on each BMI category.

	Variable	Total	Singleton pregnancy (*N* = 89)	Twin pregnancy (*N* = 70)	*p* ^∗^
Early second trimester	Normal BMI	0.096 (0.054–0.138)	0.101 (0.060–0.142)	0.092 (0.047–0.137)	0.619
	Overweight	0.107 (0.057–0.157)	0.097 (0.050–0.144)	0.114 (0.063–0.165)	0.263
	Obese	0.112 (0.060–0.164)	0.114 (0.068–0.160)	0.108 (0.044–0.172)	0.422
Mid‐second trimester	Normal BMI	0.109 (0.054–0.164)	0.106 (0.051–0.161)	0.112 (0.057–0.167)	0.948
	Overweight	0.126 (0.056–0.196)	0.095 (0.045–0.145)	0.147 (0.073–0.221)	0.003
	Obese	0.129 (0.054–0.204)	0.118 (0.063–0.173)	0.158 (0.052–0.264)	0.101
Third trimester	Normal BMI	0.119 (0.067–0.171)	0.118 (0.064–0.172)	0.120 (0.068–0.172)	0.914
	Overweight	0.135 (0.072–0.198)	0.111 (0.068–0.154)	0.152 (0.083–0.221)	0.010
	Obese	0.144 (0.074–0.214)	0.133 (0.083–0.183)	0.173 (0.072–0.274)	0.086

^∗^The results of Mann–Whitney *U* test. Results are presented as median (IQR).

**Figure 3:**
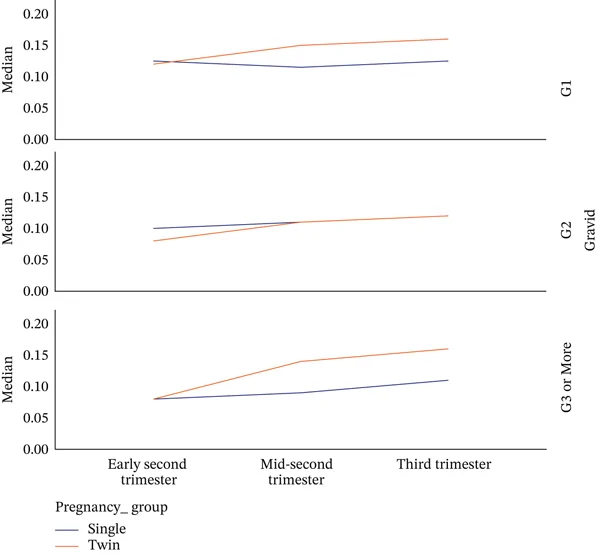
The trend of changes in median SPCR in the two study groups based on gravidity.

In Table 4, we separated the results based on gravidity and the results showed significant interaction effect of trimester‐group in Gravidity 1 (*p* = 0.012) and Gravidity ≥ 3 (*p* = 0.018); but not in Gravidity 2 (*p* = 0.136; Table 4). Comparing the median urine SPCR showed a significant difference between singleton and twin pregnancies only in women with Gravidity ≥ 3 in the mid‐second trimester (*p* = 0.016); but not in other (*p* > 0.05; Table 4, Figure 3). These subgroup findings should be interpreted with caution.

**Table 4:** Comparison of the urine SPCR in the three trimesters between the study groups based on gravidity.

	Variable	Total	Singleton pregnancy (*N* = 89)	Twin pregnancy (*N* = 70)	*p* ^∗^
Early second trimester	Gravidity 1	0.117 (0.072–0.162)	0.120 (0.073–0.167)	0.114 (0.069–0.159)	0.474
	Gravidity 2	0.105 (0.050–0.160)	0.108 (0.063–0.153)	0.101 (0.045–0.157)	0.276
	Gravidity 3 or more	0.100 (0.053–0.147)	0.097 (0.053–0.141)	0.110 (0.058–0.162)	0.460
Mid‐second trimester	Gravidity 1	0.144 (0.055–0.233)	0.122 (0.060–0.184)	0.165 (0.058–0.272)	0.151
	Gravidity 2	0.115 (0.064–0.166)	0.110 (0.059–0.161)	0.120 (0.070–0.170)	0.582
	Gravidity 3 or more	0.113 (0.054–0.172)	0.099 (0.050–0.148)	0.148 (0.080–0.216)	0.016
Third trimester	Gravidity 1	0.150 (0.069–0.231)	0.130 (0.076–0.184)	0.170 (0.073–0.267)	0.143
	Gravidity 2	0.125 (0.073–0.177)	0.120 (0.069–0.171)	0.130 (0.076–0.184)	0.495
	Gravidity 3 or more	0.132 (0.077–0.187)	0.121 (0.075–0.167)	0.158 (0.091–0.225)	0.059

^∗^The results of Mann–Whitney *U* test. Results are presented as median (IQR).

## 4. Discussion

The present study showed that women with twin pregnancies had a higher median urine SPCR value. It has to be noted that we excluded any pregnancy‐related complications, including pre‐eclampsia, and monitored the mothers′ blood pressure routinely, to investigate the urine SPCR value in uneventful pregnancies. In addition, the results showed the significant effect of twin pregnancy on urine SPCR value. In the study by Smith et al. [16], 102 women with twin pregnancies were compared with 51 singletons; they also evaluated healthy gravid patients without pre‐eclampsia and measured SPCR value in the three trimesters. The results of the study by Smith et al. [16] showed an increasing trend in SPCR values over time in all patients and documented greater SPCR values in twins than in singletons; the odds of SPCR value > 0.19 was 3.5 times higher in twins between 34 and 38 weeks of gestation. Other studies, which have measured proteinuria in 24‐h urine sample of the mothers, have also confirmed the greater values in twin pregnancies [17, 18]. Considering the association of 24‐h urine proteinuria with SPCR [20], the results of these studies can be considered in line with the general concept of our hypothesis, although the type of urine sample taken differed. Evidence has suggested SPCR as a quick and reliable test that can eliminate the need for daily 24‐h urine collection for screening and leave 24‐h urine sampling for doubtful situations [20].

The current literature available has addressed the cutoff value for considering SPCR as a predictor of pre‐eclampsia [10–14, 21]. However, the results of the present study showed that women with twin pregnancy without pre‐eclampsia had higher levels of SPCR. This finding is in line with the results of the few studies, indicating higher SPCR in women with twin pregnancies [16, 17], especially at the end of pregnancy (34–38 weeks of gestation) [16]. The present study has also determined significant differences in the mid‐second and third trimesters of pregnancy; the figures indicate an increasing trend in SPCR values over time with a greater difference in the third trimester. Review of studies on SPCR has also referred to the higher SPCR in the twin pregnancies as a result of the higher daily excretion of protein in women with twin pregnancies [12]. These findings refer to the need for estimating the SPCR cutoff and normal range for twin and multifetal pregnancies separately. Furthermore, the effect of ethnicity on SPCR has to be considered, as well, as different excretion of creatinine in the urine has been observed between different ethnicities with similar weight and height [19, 22].

Considering the difference in gravidity and BMI of the two study groups in the present study, we estimated the effect of these variables on the SPCR and the results showed the significant effect of BMI; the difference between twin and singleton pregnancies was significant in overweight and obese women after adjusting for multiple comparisons, but not in lean subjects. Smith et al. [16] have also reported BMI as a confounder of the relationship between twin pregnancy and SPCR. Association of BMI with Pr/Cr has been documented in other studies, which is possibly related to the alterations in podocyte structure or function, glomerular capillary hypertension, increased glomerular permeability to plasma proteins, and adipocyte‐derived cytokines, induced by obesity [23]. Further studies are required to determine the exact mechanism for the effect of BMI and gravidity on SPCR. This finding refers to the necessity to normalize the cutoff value based on patients′ conditions as well.

One of the strengths of the present study was studying women with uneventful pregnancy and controlling common diseases that influence the outcome. Comparison with a control group also reduced the chance of the effect of confounders on the study results. However, we cannot state that we could eliminate all factors that can influence the results. As stated, the results of this study were based on urine sample and any bias in the sampling method or laboratory analysis could influence the results of the present study; although we normalized any factor that has been shown to influence the SPCR value, such as time of sample collection [24] and urine concentration [25]. Another limitation is related to the small sample size of the study in each group, which reduced the statistical power of analysis, especially in subgroups. Additionally, although the total sample size was calculated for the primary outcome, the individual subgroup analyses (based on BMI and gravidity) may be underpowered due to the smaller number of participants in each category. This limitation may increase the risk of Type II error, and therefore, these secondary findings should be regarded as exploratory. In addition, the significant difference in baseline BMI and gravidity between the singleton and twin groups may represent a potential confounding factor, which may reflect selection bias related to the convenience sampling method; however, subgroup analyses based on BMI categories were performed to partially address this issue.

In conclusion, the higher urine SPCR values in uneventful twin pregnancies suggest the need for future large‐scale prospective studies to determine whether specific cutoff values are required for the accurate prediction of pre‐eclampsia in this high‐risk population. Further studies are required for understanding the underlying mechanism for this difference. We also documented the significant effect of BMI and gravidity on the values; therefore, a more comprehensive study is required to determine the factors associated with SPCR in women with twin pregnancy. Future studies should also measure the SPCR values in pregnancies with three or more fetuses.

## Funding

No funding was received for this manuscript.

## Conflicts of Interest

The authors declare no conflicts of interest.

## Data Availability

The data that support the findings of this study are available on request from the corresponding author. The data are not publicly available due to privacy or ethical restrictions.
